# Entrustable Professional Activities in final year undergraduate medical training – advancement of the final year training logbook in Germany

**DOI:** 10.3205/zma001278

**Published:** 2019-11-15

**Authors:** Pascal O. Berberat, Thomas Rotthoff, Christoph Baerwald, Maren Ehrhardt, Bert Huenges, Jonas Johannink, Elisabeth Narciss, Udo Obertacke, Harm Peters, Martina Kadmon

**Affiliations:** 1 Technical University of Munich, Faculty of Medicine, TUM Medical Education Centre, Munich, Germany; 2 Augsburg University, Faculty of Medicine, Augsburg, Germany; 3 University Hospital Leipzig, Medical Clinic III, Leipzig, Germany; 4 Hamburg University Medical School, Department of General Practice/Primary Care, Hamburg, Germany; 5 Ruhr-University Bochum, Medical Faculty, Department of General Medicine, Bochum, Germany; 6 University Hospital Tübingen, University Department of General, Visceral and Transplant Surgery, Tübingen, Germany; 7 Medical Faculty Mannheim of Heidelberg University, Competence Center for Final Year Medical Education, Mannheim, Germany; 8 University Medical Center Mannheim, Orthopaedic and Trauma Surgery Center, Mannheim, Germany; 9 Charité-Medical University Berlin, Dieter Scheffner Center for Medical Education and Education Research, Dean's Office of Student Affairs, Berlin, Germany

**Keywords:** final year (FY), entrustable professional activities (EPAs), competency-based training, residency

## Abstract

**Objective: **Training in the final year (FY) of undergraduate medical training currently does not adequately prepare students for the independent performance of medical professional activities after graduation. The concept of Entrustable Professional Activities (EPA) offers the opportunity for a competency-based FY training with the focus on medical professional activities.

**Methodology: **In regular meetings, the FY sub-working group of the German Medical Faculty Association (MFT), which includes representatives with clinical and didactic expertise of the Associations of Internal Medicine, Surgery and General Medicine, developed a concept for the competecy-orientated, EPA-based, FY model logbook 2.0. The selection of the units of practice was made in a cross-disciplinary, consensus-orientated discussion process based on the question which medical professional activities a young professional has to master in the inpatient or outpatient working environment.

**Results: **For the FY electives internal medicine, surgery and general medicine, a blueprint of a total of 18 comprehensive, partially interdisciplinary EPAs relating to inpatient and outpatient care contexts were developed. Each EPA was operationalised by a short description, supervision levels were attributed, and the process of transparent entrustment was determined.

**Conclusions:** The concept for a new FY model logbook 2.0 focuses on the interdisciplinary core medical professional activities in an inpatient and outpatient care context, in order to facilitate transition from undergraduate training to professional practice, and to help avoid overload, thus increasing patient safety.

## Zusammenfassung

**Zielsetzung: **Die Ausbildung im Praktischen Jahr (PJ) bereitet aktuell nicht ausreichend auf die Notwendigkeit vor, zum Zeitpunkt des Berufseinstiegs Verantwortung für die eigene ärztliche Tätigkeit zu übernehmen. Das Konzept der Anvertraubaren Professionellen Tätigkeiten (APT) bietet die Chance für eine kompetenzbasierte PJ-Ausbildung mit Fokus auf ärztlichen Handlungskompetenzen.

**Methodik: **Die Unterarbeitsgruppe PJ des Medizinischen Fakultätentags (MFT), in der Vertreterinnen und Vertreter der Fachgesellschaften Innere Medizin, Chirurgie und Allgemeinmedizin mit klinischer und didaktischer Expertise vertreten sind, entwickelte in regelmäßigen Arbeitstreffen ein Konzept für ein kompetenzorientiertes APT-basiertes PJ-Musterlogbuch 2.0. Die Entscheidung, welche ärztlichen Arbeitseinheiten Eingang finden, wurde in einem fächerübergreifenden konsensorientierten Diskussionsprozess anhand der Frage entschieden, welche ärztlichen Handlungen ein Berufsanfänger im stationären oder ambulanten Arbeitsumfeld beherrschen soll.

**Ergebnisse: **Für die PJ-Tertiale Innere Medizin, Chirurgie und Allgemeinmedizin wurde ein Blueprint von insgesamt 18 umfassenden, z.T. disziplinübergreifenden APT entwickelt, die ambulanten und stationären Versorgungskontexten zugeordnet werden können. Jede APT wurde durch eine Kurzbeschreibung operationalisiert, Supervisionsstufen wurden zugeordnet und der Prozess des transparenten Anvertrauens festgelegt.

**Schlussfolgerungen: **Das Konzept für ein neues PJ-Musterlogbuch 2.0 konzentriert sich auf fächerübergreifende zentrale ärztliche Tätigkeiten im stationären und ambulanten Versorgungskontext, um Absolventinnen und Absolventen des Medizinstudiums die Phase des Berufseinstiegs zu erleichtern, Überforderungen zu vermeiden und damit die Patientensicherheit zu steigern.

## 1. Introduction

Undergraduate medical training in the clinical phase, including the final year (FY), as well as the state licensure examinations do not always adequately prepare candidates for the independent performance of medical practice [1]. The written parts of the first and second medical state examinations (M1 and M2) focus mainly on factual knowledge, even though more and more clinically-applied topics have been included in recent years by means of the integration of case vignettes. An analysis of the written M2 examination revealed a thematic discrepancy between the surgical learning objectives in the German National Competency-based Learning Objectives Catalogue (NKLM) and the subject-related questions in the state examination [http://www.nklm.de], [2]. Comparable results were found for the questions on the common subjects of orthopaedics and trauma surgery [3]. In clinical rotations and clerkships, students are rarely given specific responsibility in patient care. At best, responsibility is limited to narrow tasks, such as taking a medical history and conducting physical examinations on patients, taking blood samples, inserting venous lines and renewing bandages. Although the oral third medical state examination (M3) takes place at the bedside, a focus on care close to clinical reality is often missing [4]. Graduates compile the patient’s history, conduct a physical examination, analyse and evaluate medical findings and, in addition, to the written report, submit an oral case report to the examination board. However, clinical decision-making in various patient-centred contexts, which is the main responsibility of a medical expert, is not reflected in the examinations. An analysis of state licensure examination questions of the Institute for Medical and Pharmaceutical Examination Questions (IMPP) between 2006 and 2012 in the subjects of internal medicine, surgery, neurology and paediatrics, revealed that just above 50% of the questions addressed aspects of clinical pattern recognition and decision-making [5]. Residents at the beginning of their careers often feel overwhelmed by the abrupt need to take responsibility for making diagnostic and therapeutic decisions in the clinical work routine [6].

At the centre of the competency-based concept of entrustable professional activities (EPA) [7], [8] is the continuous monitoring and assessment of performance in relation to defined work units in the clinical setting. The EPA concept is internationally recognized in postgraduate medical training, and also receives increasing attention in undergraduate medical education [9], [10], [11]. However, up to now, there have only been very few case studies on its implementation and the achieved effects [12], [13].

Experiences with EPAs in Germany are limited to a few individual medical schools [7], [14], [15], [16], while comprehensive approaches over all medical schools are missing. The FY seems to be particularly suitable for implementing the EPA concept, as according to the medical licensure law (ÄApprO) and the learning objectives for the FY in the NKLM, students are supposed to “deepen and extend the knowledge, skills and abilities acquired earlier in their studies”. “To this effect, they are supposed to perform their medical assignments (in line with their level of education) under the guidance, supervision and responsibility of the supervising doctor” [http://www.nklm.de], [https://www.gesetze-im-internet.de/_appro_2002/BJNR240500002.html].

### 1.1. Professional activities and the principle of entrustability

Medical competence is based on the interaction of knowledge, clinical skills and abilities, as well as personal attitudes. Epstein and Hundert suggest a definition for the clinical care context and define professional competence as “the habitual and judicious use of communica-tion, knowledge, technical skills, clinical reasoning, emotions, values and reflection in daily practice for the benefit of the individual and community being served” [17]. Medical competence becomes apparent when it is used for problem solving in variable clinical contexts. EPAs are authentic and well-defined activities which are characteristic of the medical profession and which require different competences for being mastered [18]. Thus, performance of an EPA does not only require knowledge on diseases, symptoms and procedural aspects, defined skills and communication skills, but far beyond this, these facets of competence have to be combined in order to handle a specific clinical care situation [19]. A range of competences and facets of competence underlie each EPA [20]. Carrying out ward rounds, for example, needs medical knowledge, communication skills, collaboration in a team with other health care professionals, as well as the ability to manage the patients in their specific situations.

The stepwise handover of responsibility for EPAs to students in their final undergraduate training year on the basis of their individual abilities enables them to successively train in their role as medical doctors in different clinical contexts. This facilitates the transition from undergraduate to postgraduate training with its excessive demands on novice doctors and ensures patient safety [21]. Central to this is the question which activities an individual is allowed to carry out, with how much independence and/or under which degree of supervision [22]. Thus, the EPA framework is an assessment-centred concept. The act of entrustment relies on explicit and transparent decisions on the basis of continuous observation and assessment of clinical performance, as well as on defined documented observations in clinical practice (e.g. patient registration, ward rounds, wound treatment) or work products (e.g. doctor’s letter/report). A larger percentage of the entrustment decisions is based on continuous supervision in a professional environment and a smaller percentage on additional structured observations in practice [16], [18]. The act of entrustment always involves a certain degree of subjectivity between the teaching doctor and the trainee [23]. Nevertheless, the process of the stepwise handover of responsibility linked to the specific observation offers a higher level of certainty than the current practice in which direct observation is frequently missing.

#### 1.2. The objectives of the FY in Germany

The training in the final year of undergraduate medical education before obtaining the licence to practise medicine, the FY, is intended to enable students to increasingly take on medical tasks, so that they are able to perform them independently after finishing medical school [http://www.nklm.de], [https://www.gesetze-im-internet.de/_appro_2002/BJNR240500002.html]. In reality, FY students are still given too little responsibility for performing tasks of a medical doctor [24]. Junior residents often feel overwhelmed at the start of their profession and are inadequately prepared for their daily work [25]. The stronger focus on a competency-based education or training has also shown gaps between the expectations towards medical school graduates and their performance at the beginning of the postgraduate training programme [10]. The FY-logbooks stipulated by the legislative authority for the 2013 summer semester were designed to intensify and standardise the students’ practical training; however, the established logbooks mainly list isolated practical skills, which only represent partial aspects of the more complex medical work processes. In addition, they frequently only re-offer training content from the previous clinical courses, without raising it to a higher level of complexity in accordance with the level of education, or integrating it into a specific clinical work context [26].

The EPA concept appears to be optimally suited to depict the context-related medical activities – which are not further described in the ÄApprO – and, thus, defines clear expectations for the training in the FY. In February 2013, a sub-working group (SWG) of the MFT was given the assignment to prepare a proposal for a logbook for a competency-based training in the FY on the basis of the EPA-framework. Despite the existence of a generally accessible draft, in many places the logbooks are not yet sufficiently used for training.

## 2. Concept development

### 2.1 Working group and objective

In the course of the adaptation of the ÄApprO of 2012, a final year working group of the MFT created a first logbook on a national basis for the mandatory electives of the final year [27], which was made available to all the medical faculties in the autumn of 2012. Many faculties used this template, adapted it to their own needs and developed on this basis logbooks for other electives [28]. 

The SWG FY-Logbook pursued the aim to further develop this basis logbook. The resulting concept for a new FY model logbook 2.0 should serve as the basis for a more competency-oriented FY, with a focus on more complex medical professional skills. It focuses on the daily work routine of a new doctor during the first months of residency. Furthermore, according to the recommendation of the Master Plan Medical Studies 2020 [29], general medicine should be considered in addition to the compulsory disciplines of surgery and internal medicine.

Under the direction of the authors, Pascal Berberat and Martina Kadmon, the SWG was formed, in which clinically-experienced experts from the three disciplines are represented. Two of the authors come from primary care, all the others (as part of their faculty responsibilities) had close connections with those who are responsible for the FY training in teaching hospitals. During the project, one of the authors was working in the medical faculty that monitors the entire clinical training in collaboration with municipal and ecclesiastical hospitals. Thanks to this, the perspective of all the institutions that are involved in the FY was considered in the concept development. All members of the SWG FY-Logbook are recognised as representatives of the medical professional associations and, furthermore, they have special qualifications as lecturers in FY training and curriculum development.

The SWG FY-Logbook has met 17 times in the last six years. During this working period, a core group remained stable; individual members left and were replaced by others, and an adequate representation of the three disciplines was always maintained. This ensured an in-tensive, interdisciplinary and consensus-oriented work method, with the agreement of all the participating disciplines. 

#### 2.2 The creation of inter-disciplinary and specialised EPAs for the FY 

The decision as to which medical work units should be integrated into the EPAs was taken in an interdisciplinary and consensus-oriented voting process during the first working group meetings. The guiding question for this decision was: “What medical activities should a starting professional on his or her first day of employment be able to master in the various clinical environments (ward, outpatient clinic, OP/surgery, functional areas)?” Once this basis had been established, the content of a model EPA was formulated, which was also agreed in a joint meeting. On the basis of this model EPA, the rest of the EPAs were formulated discipline-specific, mainly in a circulatory process, and finally approved in working group meetings.

For the decision on the entrustment levels, the working group orientated itself on the following guidelines and principles:

1. The training in the FY, and in the first two residency years, was taken as a continuum, so that for the EPAs in the FY, no comprehensive transfer of responsibility in the sense of entrustment level 5 (“may provide supervision to junior trainees”) [18] has to be achieved (see also table 1 (Tab. 1)).
2. The EPA should be developed in close conjunction with the NKLM.
3. In line with the recommendations of the Master Plan Medicine 2020 [29], the integration of general medicine should especially strengthen the area of outpatient care, within the framework of the FY training.

A crucial challenge in the creation of EPAs was to determine the granularity, which can range from smaller tasks for students, to the complex activities at a specialist doctor level [22], [23]. As students in the FY increasingly undertake real medical tasks and have to perform them safely and independently on patients at the time of starting their careers [http://www.nklm.de], [https://www.gesetze-im-internet.de/_appro_2002/BJNR240500002.html], EPAs were formulated as rather comprehensive units of activity (e.g. inpatient care for a post-operative patient), but also, as more specifically defined medical procedures (e.g., “conducting a vaccination counselling”). Independent of granularity, in addition to knowledge and practical skills, all EPAs address advanced skills, such as communication skills in conversations with patients and relatives, but also attitudes which are reflected in the overall behaviour, e.g. in hygiene habits. However, the respective requirements of the acting individual are very different in their degree of complexity. Very comprehensive EPAs carry the risk of not being fully entrustable to students due to inadequate prerequisites. Very narrowly defined EPAs run the risk of breaking down medical practice into individual work processes and – similar to the effect of an excessive number of learning objectives – making the overview and handling in clinical training more difficult [30]. After extensive and sometimes controversial discussions, the SWG decided to define comprehensive, not too fine-grained EPAs, in order to do justice to the integrating and conclusive character of the FY in the context of the medical school, and to the continuum of training in residency. The connection to the NKLM was ensured by the fact that for each EPA the underlying learning objectives in the domains of knowledge and clinical skills and, across all EPAs, overarching learning objectives in the “attitude domain” were for-mulated, which are defined in the NKLM as the basis for the core curriculum in medicine. In addition, there is the possibility of defining less comprehensive EPAs for the study phase prior to the FY, that build on and complement one another (“nested EPA”), which then lead to the less extensive “core EPAs” in the FY [11]. Thus, the SWG FY-Logbook deliberately took a different path than other current national and international initiatives that try to embed EPAs in medical school [9], [10], [11], [16], [18], [31]. In this way, a new approach to training in the FY, with a stronger professional orientation and with the integration of the students into a team of employees from the various health professions, was to be achieved.

An EPA blueprint was developed for the compulsory internal medicine and surgery electives, as well as for the general medicine elective, to which outpatient and inpatient care contexts could be assigned (see table 2 (Tab. 2)). When defining EPAs, the interdisciplinary discussion revealed significant overlaps in the medical activities of the three selected disciplines. For example, the EPA of “Preparation and realisation of a release/discharge plan” plays a central role in both internal medicine and in surgery. The consultation of a patient who has an acute medical condition, or who needs immediate treatment, is a central activity in the outpatient care sector of all disciplines. The differences between the disciplines will, of course, exist in relation to the specific and necessary disease-oriented knowledge or defined manual skills, but the competences underlying EPAs are not primarily discipline-related, but context-related. This also makes it clear to the students, that main professional medical activities are relevant across disciplines and, therefore, are not only to be learned in their potentially aspired discipline.

There is a total of seven EPAs described for the surgery elective, six EPAs for the internal medicine elective and five for the elective general medicine (see table 2 (Tab. 2)) (example, see table 3 (Tab. 3)). These are categorised according to the two central learning environments of inpatient and outpatient care and contain both interdisciplinary and discipline-specific competences. Accordingly, some EPAs will be used in different electives of the FY, others can be trained exclusively in one elective. For each EPA, a brief description has been provided which contains the discipline, learning environment, a short content description, the recommendation for the entrustment level, as well as the limitations on its use. The more detailed operationalisation represents the knowledge and clinical skills required for the medical activities and is based on the learning objectives of the NKLM. It can serve both medical schools and students as a guideline for clinical education in preparation for the FY.

#### 2.3 Development through supervision levels

The entrustment of medical activities under gradually decreasing levels of supervision is the core of the EPA framework concept. The supervision levels show how well and how independently a FY student is able to perform a professional activity [8]. The supervision levels for EPAs were defined in accordance with the AMEE Guide No. 99 [18] (see table 1 (Tab. 1)).

To what extent, the differentiation of entrustment levels in clinical practice is practicable and feasible, must be shown in the actual implementation.

In accordance with the legal framework, FY students should learn core medical activities in such a way that they at the best perform them under indirect supervision (entrustment level 3a-c) or, at the end of the FY, reach level 4 ('allowed to practice EPA unsupervised, with dis-tant supervision). In the context of the continuum between medical school and residency, they should in the first months of residency increasingly assume responsibility and certainly reach confidence levels 4 and 5 (“can provide supervision to junior trainees in performing this activity”) [7], in order to be ready and prepared for stand-by duty. Such an “ability to serve” is a classic EPA, historically grown in the areas of healthcare. 

#### 2.4 Deliberate decision of entrustment

The entrustment of an activity is clearly defined from level to level and is based on an explicit and transparent decision after adequate discussion in the responsible and (preferably) inter-professional team [32]. This takes place on a regular basis (e.g. every four weeks), is documented in writing and explained to the students in feedback discussions. The decision is essentially based on continuous supervision by supervising doctors, as well as by all the people involved in the clinical setting, from both the medical and nursing professions (360° feedback). The continuous observation involves numerous observation situations in the daily work which does not always concern the entire medical activity, but only parts of it. The multiple observation situations, by different observers, result in an overall assessment, allowing a safer entrustment decision to be taken, than if only one person would observe. This kind of continuous observation appears well-suited for an EPA, such as the “inpatient care of a patient”, i.e. only decisions based on explicitly documented, structured observations and standardised assessment situations (see 1.1).

In addition to the continuous observation, the following types of targeted evaluations have been defined [33], and individual examples have been developed as the basis for entrustment decisions [34], [35], [36] [https://medizinische-fakultaeten.de/studium/themen/glossar/praktisches-jahr/]:

1. Evaluation of working products, e.g. admission form, documentation of patient care, preparation of a treatment plan, release/discharge letter.
2. Evaluation of case-based presentations / discussions, e.g. presentations about patients at indication discussions, colloquia, tumour conferences, patient hand-overs)
3. Evaluation of practice observations (e.g. ward rounds, patient admission, release/discharge interview, telephone calls with follow-up attending doctors, wound treatment)

Possible structured assessment formats are MiniCEx (Mini Clinical Examination) e.g. for ward rounds, Encounter Cards, e.g. for patient admission, and DOPS (Direct Observation of Procedural Skills), e.g. for wound treatments. But also the previously mentioned parameters in the form of specific work results, case-based discussions and practical observations can be seen in daily clinical practice and do not require a complex preparation process. The guidelines for a competency-based FY model logbook 2.0 provides elaborate handouts for individual EPA assessments [34], [35], [36].

A fundamental feature of the EPA structure is that the entrustment processes, which continuously and implicitly take place in clinical practice between the experienced and lesser experienced doctors, are made explicit. As these decisions are made in a consensus between all those involved in the clinical- and training context (360° feedback) and are based on specific observations, they make a significant contribution to patient safety and help to avoid situations in which FY students (and later also starting residents) feel either under- or overwhelmed. An important prerequisite for this is an institutionalised professional and inter-professional error culture. The extent to which assessments of supervisors from previous electives are adopted is left to the respective supervisor and depends, among other things, on the extent to which the EPA concept can be implemented throughout a hospital in the context of the FY training. Of particular importance, however, is also the perceived trustworthiness and reliability of the student [18], [32]. The principle of trust and entrustment requires the following three fundamen-tal elements:

1. Integrity, i.e. the performer’s good intentions and honesty,
2. the student’s reliability, i.e. the ability to work consistently and predictably, and
3. the student’s sincerity, i.e. the recognition of the student’s own boundaries and the willingness to ask for support timely.

The transparent decision for a comprehensive granular EPA could lead to a “portioned/partial” entrustment of the respective EPA. In everyday clinical practice, this is definitely possible and also desirable (as part of the so-called “ad hoc entrustment”) [16], [18]. However, the written assessment at the pre-determined intervals (see above) should include a clear and unambiguous definition at which level of supervision an EPA may be performed without the risk of endangering the patient. The result of the assessment is transparently documented in the log-book and communicated to the student in a structured feedback discussion. In order to support the student’s continuous competency development, the structure of these discussions should contain the aspects of “Feedback – Feed-up – Feedforward” [37]. In the “feedback” phase, the FY student’s self-reflection on the EPA is an essential element. In addition, the supervising doctor explains the 360° feedback of the supervisors involved. During the “feed-up” phase, the objectives to be achieved in the next observation period are jointly defined in order to encourage the student’s self-directed learning. A reflection takes place in the “Feed-forward”, with which concrete steps and activities this objective can be achieved. In order to effectively use the potential of the EPA concept, a comprehensive feedback culture needs to be integrated into the FY training. This concerns both the intensification of the feedback competency of the FY supervisors in the faculty development programmes and the use of feedback by the FY students. In particular, their self-reflection skills must become an integral element of the FY training.

## 3. Guidelines for a competency-based FY model logbook 2.0

Guidelines for a competency-based FY, based on the EPA framework, were developed for the two compulsory electives of internal medicine and surgery, as well as for the optional elective, general medicine [34], [35], [36]. 

A condensed introduction first explains the objectives and the current legal basis of the FY, as well as the basic principles of EPAs. Furthermore, the application of EPAs in practice and, in particular, the entrustment process, the conducting of feedback discussions and documentation of the learning process are discussed in detail. This is congruent for the disciplines internal medicine and surgery, whereas in general medicine, in the ambulatory context, only a few adjustments have been made. In the second part, the discipline-specific EPAs are displayed and then operationalised in detail in the respective individual EPA descriptions. Finally, the third part contains documentation samples for the feedback discussions and for selective observations. These guidelines form the basis for adapting university-specific logbooks to the new EPA-based FY concept. At the same time, they serve to explain the concept in theory and practice to the attending doctors, as well as the students, as pragmatically and precisely as possible.

## 4. Discussion

During the last few years, the MFT’s SWG FY-Logbook has developed a comprehensive concept for a competency-oriented training in the FY in accordance with the principle of “Entrustable Professional Activities” (EPA). The model, which was originally developed for postgraduate training, was adapted to the final workplace-based phase of medical school. In this process, not just the legal framework conditions were considered, but also other current developments in medical education in Germany; in particular the NKLM and the requirements of the so-called Master Plan for Undergraduate Medical Training 2020 were taken into account.

In accordance with the competency-based approach, the EPA concept and the concept for the FY model logbook 2.0, for the mandatory internal medicine and surgical electives, as well as the optional elective general medicine, focus on interdisciplinary central medical profes-sional activities in a clinical and outpatient context. The aim is to integrate knowledge, clinical skills and attitudes into the performance of basic clinical activities – away from individual practical skills to important, but also complex professional activities. The concept shifts from the pure assessment of competence based on the quantity of procedures performed towards the assessment of the qualitative overall impression, which is underpinned by continuous supervision and standardized observations.

Open questions mainly concern the implementation of the EPA concept and will be discussed here:

1. Is the comprehensive granularity of the EPA concept, which aims to make comprehensive medical activities visible across disciplines, feasible in the training practice for students and supervisors? Other working groups defined significantly more fine-granular EPAs. EPA 1.1, in the work of Holzhausen et al., which was also formulated for the graduate level [11] (“Taking a patient history, carrying out a physical examination and summarising results in a structured way”) is less comprehensive when compared, for example, to the EPA defined here (“Inpatient admission of a patient with acute symptoms”). However, the defined individu-al activities are also included in the comprehensive EPA, i.e. “nested”. The question of which granularity is the most suitable for the graduate profile, will have to be shown by practice. Ten Cate et al. [18], [38] discussed the advantages and disadvantages of comprehensive and fine granular EPA in detail, especially with regard to the act of entrustment: Whilst fine granular EPAs appear to be more clear and easier to entrust, the comprehensive granular EPAs are more oriented towards the daily work of a doctor and their entrustment means a larger step in the development of competencies.
2. How can the act of entrustment be ensured on a regular basis and in a quality that is effective for learning, and how do such decisions affect training and clinical practice? The answer to this question will determine whether the EPA framework will be put into practice. It seems important for the purpose of argumentation, that implicit processes in education and training, that at least in part happen regularly already now, must only be made explicit and transparent. The group around Ten Cate made clear that, in the clinical day-to-day work, decisions in the sense of entrustment are made over and over again (‘entrustment decision’), mostly spontaneously in a 1 to 1 training situation, and according to the specific context (so-called “ad hoc entrustment decision)” [18], [38]. The EPA concept now offers the opportunity to summarise these on a regular basis and to make and communicate explicit, transparent decisions – a so-called “summative entrustment decision”. This entrustment is based on the assessment of multiple observers, who revert to multiple observation moments and various sources [18], [38]. The understanding and acceptance of the EPA concept by supervisors and students is crucial. On the one hand, the fact that the EPAs are defined on the basis of clinical core activities promotes mutual acceptance [7], [8]. On the other hand, teachers may not take the high demands made by explicit, continuous and supportive supervision combined with regular and structured feedback as a matter of course. The explicit assumption of responsibility and the required regular self-reflexions will also initially be a high demand for students. For this to succeed, a comprehensive feedback culture must be integrated into the FY training. This concerns both the intensification of the feedback competency of the FY supervisors in the faculty development programmes and the use of feedback on the part of the FY students. Especially their competence in self-reflection must become an integral part of the FY training [16].
3. How can this PJ curriculum be designed to make the connection to clinical education before the FY, and to subsequent postgraduate training? As already mentioned, the entrustment of comprehensive granular EPAs at the graduate level in the FY, must be gradually prepared by smaller (“nested”) EPAs in the clinical education in the second stage of medical school EPA [18]. The practical realisation of an uninterrupted chain of entrustment across various teaching environments and training phases (e.g. examination courses > clinical courses > clinical clerkships > FY electives) still poses an unresolved challenge. One option could be the consistent use of portfolios throughout the entire studies [16]. This is based on the principle of self-directed learning, in which students document and drive their own learning process in a holistic and self-responsible manner [38]. The same challenge arises for the continuity between medical school and residency; the EPAs described here would have to be relatively quickly entrusted to level 4 after entering the medical profession [7]. As it is also unlikely that logbooks will be passed on from the university to the residency provider in the future, practical solutions need to be developed in agreement between the universities and medical associations so that here, too, the chain of trust is not interrupted. Last, but not least, the oral-practical examination in the third section of the medical licensure examination (M3) is of central importance for the competency-based training in the final year and, therefore, also for the acceptance of the EPA concept.
4. How can the new form of explicit observation and as-sessment of performance be meaningfully linked to M3? The current coordinated process of revising the NKLM and the subject catalogues also includes the definition of a graduate profile using the EPA framework. A basis for this is the result of the work conducted by the MFT’s SWG FY-Logbook on the current EPAs, as well as the preparatory work at the Charité Berlin [11], which are included and in agreement with the working group “graduate profile” of the IMPP. Consensus on such a graduate profile can influence the orientation of the M3. And this, in turn, will then act as an effective driver for the realisation of competency-based training in the FY.

These open questions will have to be empirically explored and further discussed in the course of practical testing, with the justified hope that the EPA-based training in the FY will facilitate the professional start for medical graduates, help to avoid excessive demands, and thereby increase patient safety.

## Competing interests

The authors declare that they have no competing interests. 

## Figures and Tables

**Table 1:**
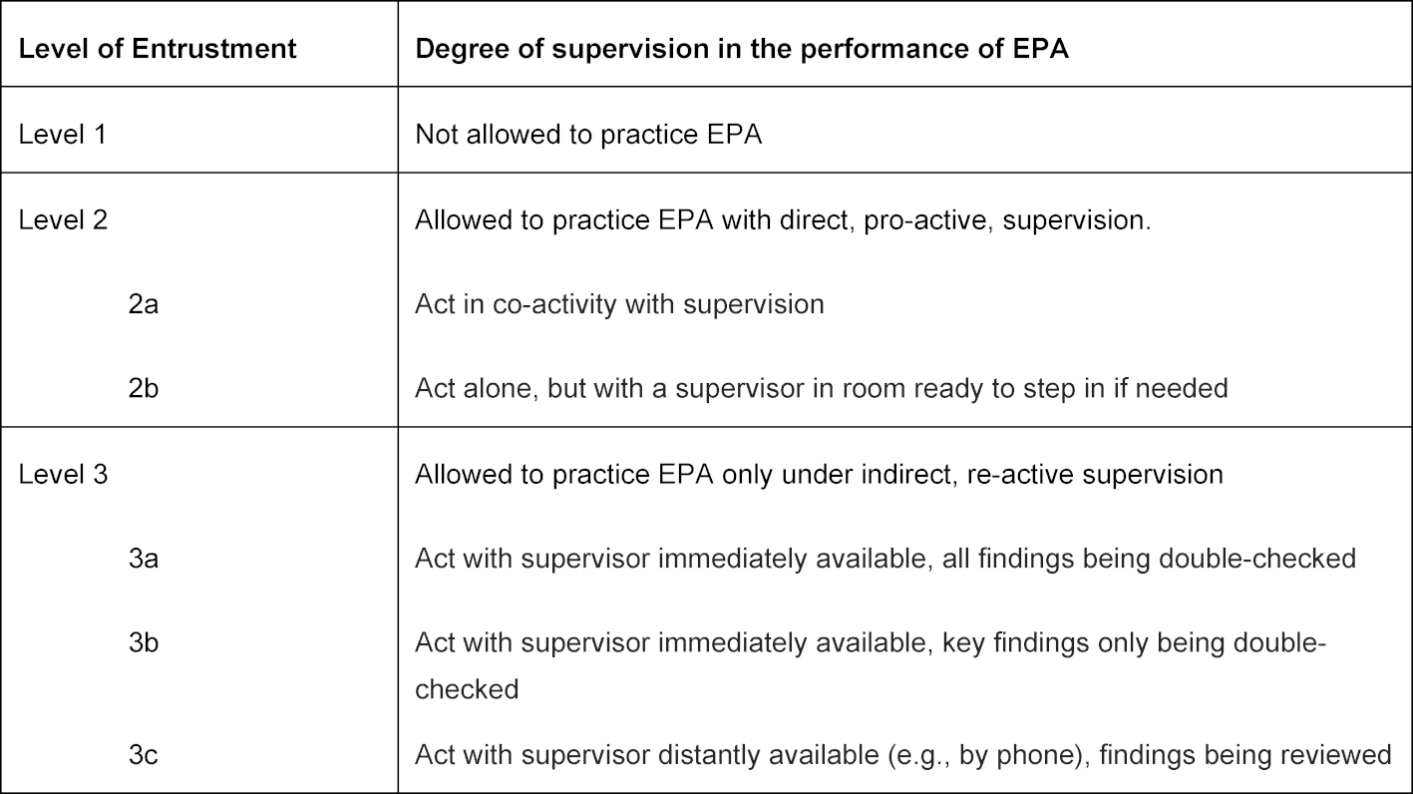
Levels of supervision and entrustment of medical activities in the FY [9, 18]

**Table 2:**
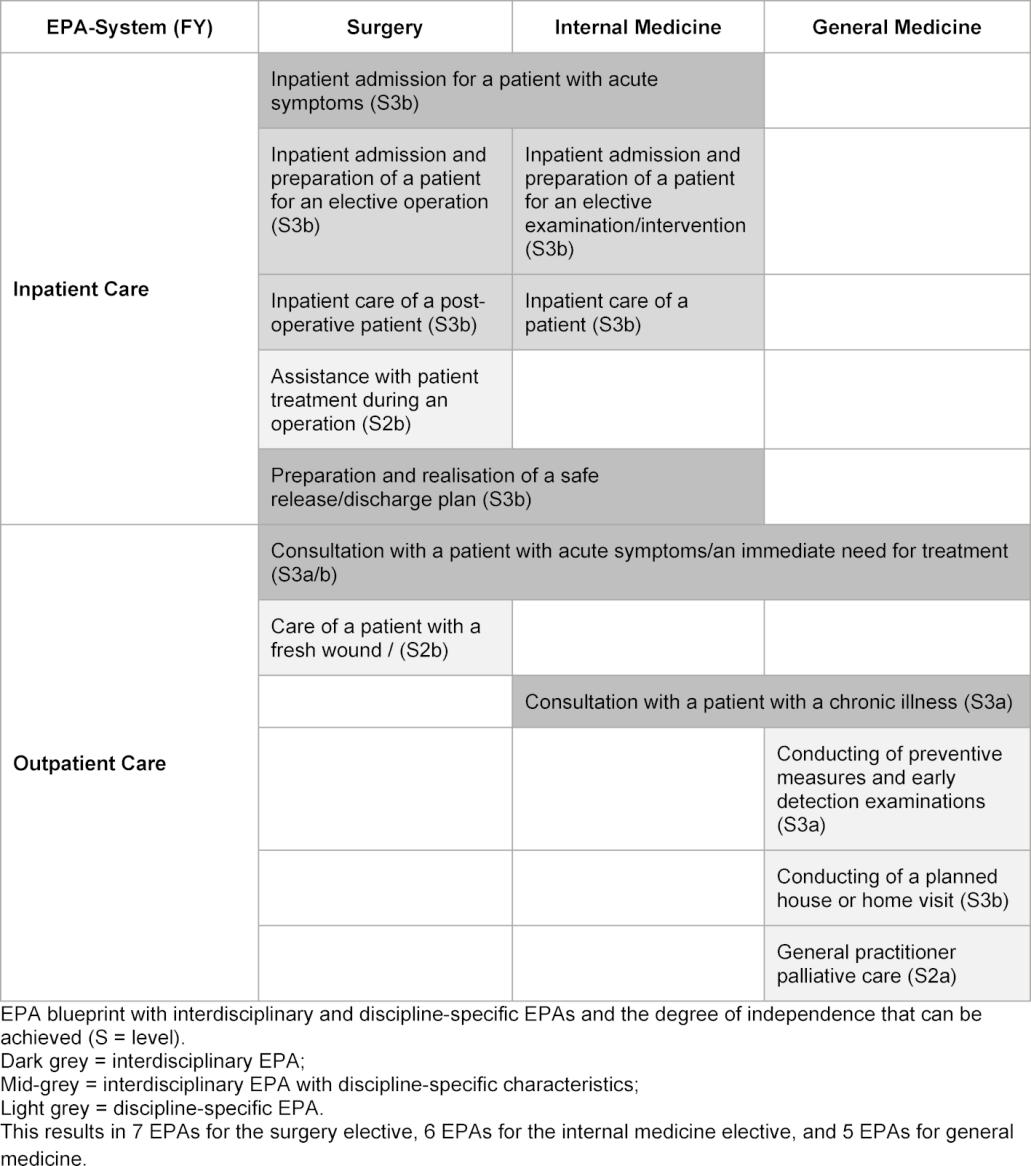
EPA-Titles (FY)

**Table 3:**
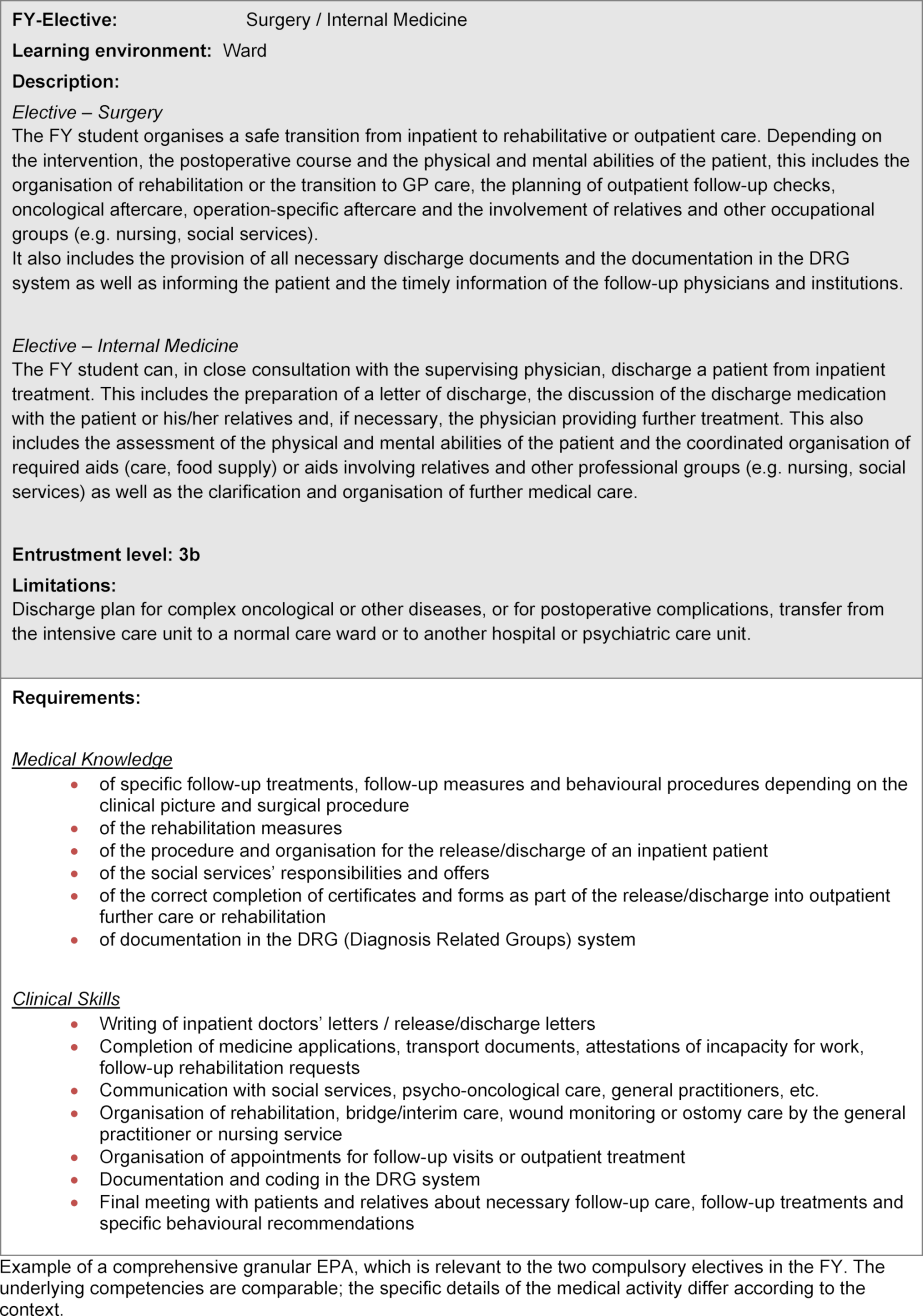
Example EPA: Preparation and realisation of a safe release/discharge plan
